# The mediating role of biological age in the association between dietary index for gut microbiota and sarcopenia

**DOI:** 10.3389/fimmu.2025.1552525

**Published:** 2025-03-21

**Authors:** Jingyuan Zhang, Jun Guo, Jing Zhang, Heng Liu, Lin Zhou, Chi Cheng, Hong Cao

**Affiliations:** ^1^ Department of Traumatic Orthopedics, Renmin Hospital, Hubei University of Medicine, Shiyan, China; ^2^ The Second Department of Infectious Disease, Shanghai Fifth People’s Hospital, Fudan University, Shanghai, China; ^3^ Center of Community-Based Health Research, Fudan University, Shanghai, China; ^4^ Department of Urology, Renmin Hospital, Hubei University of Medicine, Shiyan, Hubei, China

**Keywords:** DI-GM, sarcopenia, biological age indicators (PhenoAge, HD, KDM), gut-muscle axis

## Abstract

**Background:**

Dietary Index of Gut Microbiota (DI-GM) is a newly proposed comprehensive metric for assessing dietary quality in relation to gut microbiota composition. Alterations in muscle structure are closely linked to DNA methylation-based biological age assessments and individual dietary patterns. However, a systematic investigation of the interrelationships among DI-GM, biological age, and sarcopenia remains lacking. We hypothesize that consuming foods beneficial to the gut microbiota may help mitigate the risk of sarcopenia by slowing the aging process.

**Methods:**

This study analyzed data from NHANES 2007–2018. DI-GM was calculated using two 24-hour dietary recall datasets. Sarcopenia was assessed via dual-energy X-ray absorptiometry (DXA). The association between DI-GM and sarcopenia was evaluated using multivariate logistic regression, subgroup analysis, and restricted cubic splines. This study also investigated the potential mediating effects of three biological age indicators: the Klemera-Doubal Method (KDM), PhenoAge, and Homeostatic Dysregulation (HD).

**Results:**

An increase in DI-GM score was significantly associated with a reduced risk of sarcopenia (OR: 0.87, 95% CI: 0.82–0.94).The risk of sarcopenia was significantly lower in the highest quartile group (Q3) (OR: 0.25, 95% CI: 0.11–0.58). The three biological age-related indicators (KDM, PA, and HD) partially mediated the association between DI-GM and sarcopenia, with PhenoAge showing the highest mediation proportion at 30.6%.

**Conclusion:**

A higher DI-GM score was significantly associated with a reduced risk of sarcopenia. PhenoAge, HD, and KDM demonstrated significant mediating effects, with PhenoAge showing the highest mediation proportion.

## Introduction

Sarcopenia is characterized by the gradual loss of muscle mass, muscle strength, and physical function. It not only significantly affects the daily living abilities of older adults but also increases the risk of falls and fractures. In fact, the prevalence of sarcopenia in middle-aged and elderly populations (60–70 years) is 5–13%, while this proportion rises significantly to 11–50% in individuals aged 80 years and above (1, 2). With the increasing prevalence, sarcopenia has led to a significant rise in the demand for healthcare and caregiving services. The study showed that the average annual hospitalization cost per person with sarcopenia in the United States is about $2,315.7, while in the UK, the figure is as high as £2,707 (3). It is estimated that if the prevalence of sarcopenia could be reduced by 10%, the US healthcare system could save approximately $1.1 billion per year (4). Currently, resistance training is the main treatment for sarcopenia, but patients’ poor compliance limits its effectiveness. Given the limited efficacy of drug treatment, adjusting dietary structure and supplementing nutritional supplements may become a promising auxiliary intervention strategy.

Aging is a multifactorial biological process that is closely related to a variety of chronic diseases and functional degeneration. Aging not only leads to a decline in muscle mass and physical performance, but also significantly affects the diversity and functionality of the gut microbiota. At the same time, aging assessment can be quantified through different biomarkers. For example, the Klemera-Doubal Method (KDM), a biomarker-based method for assessing physiological age, the phenotypic age (PhenoAge) that reflects the risk of death, and the Homeostatic Dysregulation (HD) index that measures the degree of deviation from the balance of the physiological system. These indicators can reflect the degree of aging of an individual in different aspects. Compelling evidence suggests that intervention in the gut-muscle axis may reverse the sarcopenic phenotype (5). Li et al. reported that Lactobacillus and Bifidobacterium supplementation significantly improved muscle metabolism, reduced inflammation, and enhanced mitochondrial function in aged mice (6). A randomized controlled trial showed that continuous probiotic supplementation can improve muscle strength and physical function in older adults (7). However, Zhang et al. found that most interventions studied to date have focused on a single bacterial community, and the effects of a single bacterial community may change due to interactions with other microorganisms (8). Therefore, the effects of different probiotic supplements are not yet sufficient to be promoted to a wide range of people. Recent studies have begun to adopt a “holistic dietary approach”, but the most commonly used Healthy Eating Index (HEI), Alternate HEI (aHEI), Mediterranean Diet Score (MDS), and Dietary Approaches to Stop Hypertension (DASH) have been inconsistently associated with indices of gut microbiota diversity and richness (9–12). Therefore, Bezawit E Kase et al. developed an index for comprehensive assessment of gut microbiota diversity - DIGM as a standardized tool for evaluating diets that promote a healthy gut microbiota.

Existing research has primarily examined the association between the Dietary Index of Gut Microbiota (DI-GM) and metabolic as well as mental health (13). However, its relationship with sarcopenia remains largely unexplored. Utilizing data from NHANES (2007–2018), this study systematically investigates the association between DI-GM and sarcopenia for the first time. Furthermore, it explores the mediating role of three aging indicators—PA, HD, and KDM—in this relationship.

## Methods

### Data sources and study population

**Figure 1:**
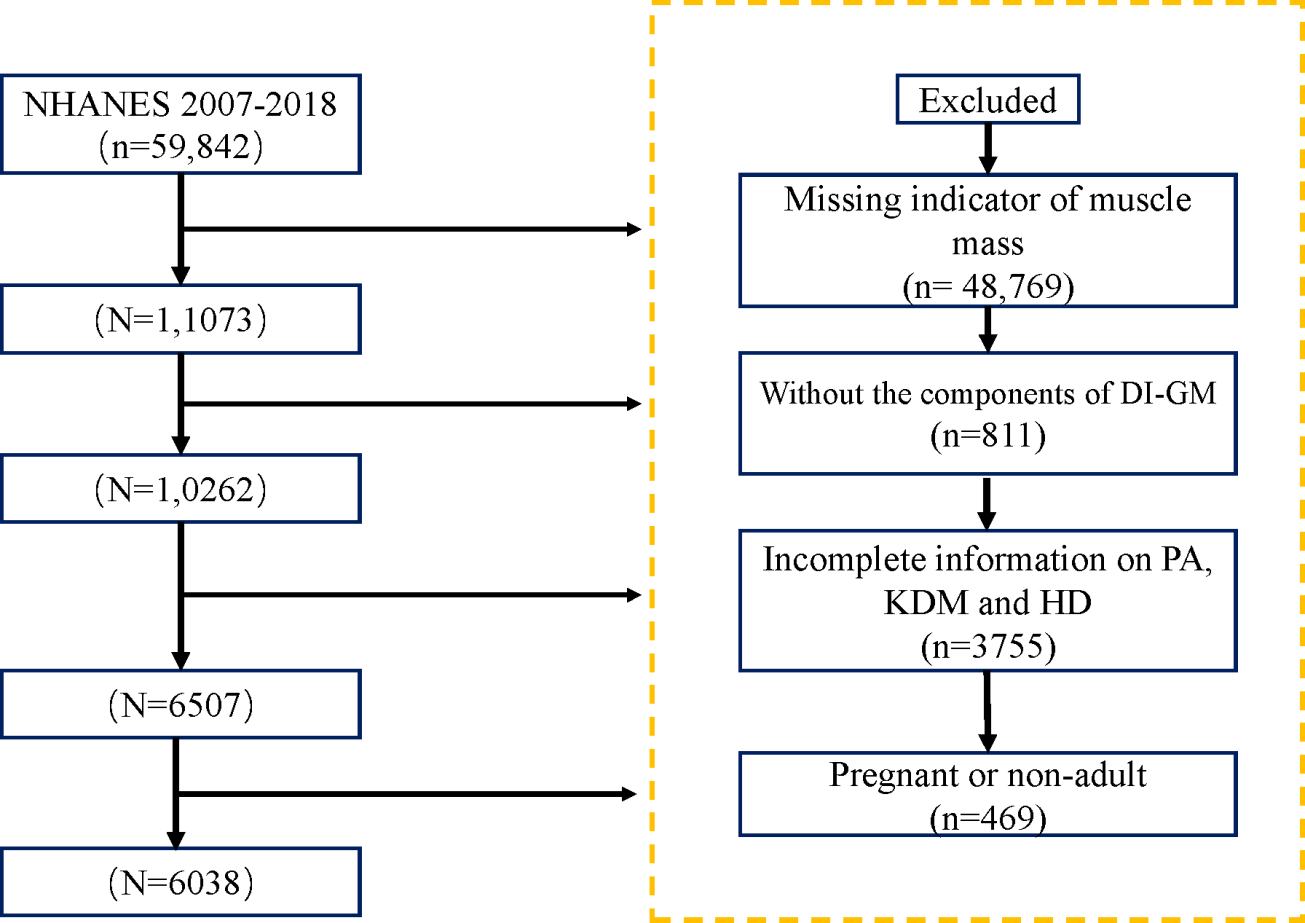
Study population inclusion process.

The National Health and Nutrition Examination Survey (NHANES) is a major health and nutrition survey conducted by the National Center for Health Statistics (NCHS), which operates under the Centers for Disease Control and Prevention (CDC). Researchers can access the survey questionnaires, technical documentation, and analysis tools through the official website: https://www.cdc.gov. The data analyzed in this study were derived from NHANES 2007–2018, ultimately including 6,038 participants who met the specified criteria, (**Figure 1**).

### Exposure assessment

Dietary data in the NHANES were collected through two 24-hour dietary recall interviews (24HR). The first interview was conducted in the Mobile Examination Center, where participants recalled and reported all food and beverage intake from the previous 24 hours (14). The second interview was administered via telephone 3 to 10 days after the initial interview. To enhance data reliability, records with extreme daily caloric intake (less than 500 kcal or more than 6000 kcal) and extreme BMI (below 15 kg/m² or above 65 kg/m²) were excluded. All reported dietary intake data were coded using the Food and Nutrient Database for Dietary Studies (FNDDS) from the United States Department of Agriculture (USDA) (15). In this study, dietary data used to construct the DI-GM were averaged across the two recall interviews. This approach was implemented to minimize individual recall bias and account for potential variations between interview days.

Following the successful development of the Dietary Inflammatory Index (DII) and the Healthy Eating Index (HEI), Bezawit E. Kase developed a scoring system to assess gut health—DI-GM (16). The DI-GM score incorporates 10 gut-health-promoting foods and 4 foods detrimental to gut health. The DI-GM assigns scores based on sex-specific medians or fixed thresholds for intake. A score of 1 is given if the intake of beneficial components is above the median or if the intake of detrimental components is below the median. The total score ranges from 0 to 14. A higher DI-GM score indicates a dietary pattern with more significant health effects on gut microbiota. The specific calculation details and scoring criteria have been thoroughly described in the study by Bezawit E. Kase et al (16). In addition, the relevant calculation methods can also be found in the **Supplementary Materials**.

### Outcome variable

This study used dual-energy X-ray absorptiometry (DEXA) to measure body composition, including fat mass, lean body mass, total body fat percentage, lean body mass percentage, and appendicular skeletal muscle mass (ALM). According to the FNIH criteria, sarcopenia is defined as ALM/BMI < 0.789 for men or ALM/BMI < 0.512 for women (17).

### Mediating variables

This study assessed biological age using the Klemera and Doubal Model (KDM), PhenoAge (PA), and Homeostatic Dysregulation (HD). Eleven blood biomarkers were used to calculate the relevant indicators: alkaline phosphatase, total cholesterol, uric acid, albumin, creatinine, glycated hemoglobin, white blood cell (WBC) count, lymphocyte percentage, mean cell volume, blood urea nitrogen, and red cell distribution width (18, 19). The collection and processing of serum samples were conducted by the Mobile Examination Centers. All algorithms were trained using biomarker data from NHANES III (1988–1994), as developed by Nakazato et al (20). The related code is available via the R package “BioAge” at: https://github.com/dayoonkwon/BioAge.

### Covariates

Based on previous studies, this study included potential confounding variables that may influence sarcopenia (21). Demographic variables included age, sex, race/ethnicity, education level, and marital status. Socioeconomic factors included the PIR. Additional covariates included hypertension, diabetes, cardiovascular disease (CVD), smoking status, alcohol consumption, BMI, and moderate physical activity levels.

Smoking status was classified into three categories: current smokers (≥100 cigarettes in a lifetime and currently smoking), former smokers (≥100 cigarettes in a lifetime but no longer smoking), and never smokers (<100 cigarettes in a lifetime or never smoked) (22). Diabetes was defined based on either a physician diagnosis or a fasting blood glucose level ≥126 mg/dL. In addition to diabetes, this study included prediabetes, which was categorized as either impaired fasting glucose (IFG) or impaired glucose tolerance (IGT) (23). Hypertension was determined by a previous physician diagnosis, current use of antihypertensive medication, or an average of three blood pressure measurements ≥140/90 mmHg. Stroke was classified as either present or absent. CVD was defined as a history of congestive heart failure, coronary artery disease, angina, or myocardial infarction.

Alcohol consumption was categorized into five groups based on lifetime alcohol intake and binge drinking frequency (24). Never drinkers were defined as individuals who consumed fewer than 12 alcoholic drinks in their lifetime. Former drinkers included those who had consumed at least 12 alcoholic drinks in a single year but had not consumed alcohol in the past year or had not consumed alcohol in the past year but had a lifetime history of at least 12 drinks. Mild drinkers were individuals who consumed alcohol occasionally, with category 1 frequency for females and category 2 for males. Moderate drinkers were those who consumed alcohol with category 2 frequency for females and category 3 for males or engaged in binge drinking between ≥2 and <5 episodes. Heavy drinkers were individuals who consumed alcohol frequently, categorized as category 3 for females and category 4 for males, or those who engaged in ≥5 binge drinking episodes.

### Statistical analysis

This study conducted weighted analyses using primary sampling units (PSU) and stratification variables (Strata), with multi-cycle weight adjustments (weights divided by 6). In this study, continuous variables were presented as means with standard errors, while categorical variables were expressed as counts and percentages. Differences among categorical variables were assessed using the chi-square test, while differences among continuous variables were evaluated using the Wilcoxon rank-sum test.

The association between DI-GM and sarcopenia was evaluated using multivariate logistic regression in this study. This study used odds ratios as statistical indicators to evaluate the impact of DI-GM on the risk of sarcopenia. Model 1 was unadjusted for covariates. Model 2 adjusted for demographic variables. Model 3 adjusted for age, sex, race, education level, marital status, PIR, BMI, smoking, alcohol consumption, diabetes, hypertension, CVD, and physical activity status. The potential nonlinear association between DI-GM and sarcopenia was analyzed using RCS curves with the ggrcs package. Additionally, subgroup analyses were conducted based on factors such as age, sex, education level, BMI, alcohol consumption, CVD, and hypertension.

To evaluate the mediating effects of KDM, PA, and HD in the relationship between DI-GM and sarcopenia, we conducted a causal mediation analysis. The “mediation” package in R was used to quantify the direct effects, indirect (mediated) effects, and total effects of KDM, PA, and HD in this association. In this study, DI-GM was designated as the independent variable, sarcopenia as the dependent variable, and KDM, PA, and HD as potential mediators. To construct a nomogram and assess the predictive performance of DI-GM for sarcopenia, participants were randomly divided into a training set (70%) and a validation set (30%). A Neural Network model was employed to identify the most influential predictive variables. The predictive accuracy of DI-GM in relation to sarcopenia was subsequently evaluated using a receiver operating characteristic curve.

All statistical analyses were conducted using R software (version 4.2.3) and EmpowerStats (version 4.1). A P-value < 0.05 was considered statistically significant.

## Results

### Characteristics of participants

A total of 6,038 participants were included in this study and divided into four groups based on the quartiles of DI-GM. The mean age of the participants was 40.20 ± 13.78 years, and participants with higher DI-GM scores had a lower proportion of sarcopenia. Additionally, participants with higher DI-GM scores exhibited lower BMI, smoking rates, and alcohol consumption rates, along with higher proportions of physical activity and non-Hispanic White ethnicity, **Supplementary Table S1**.

### Association of DI-GM with sarcopenia

**Figure 2:**
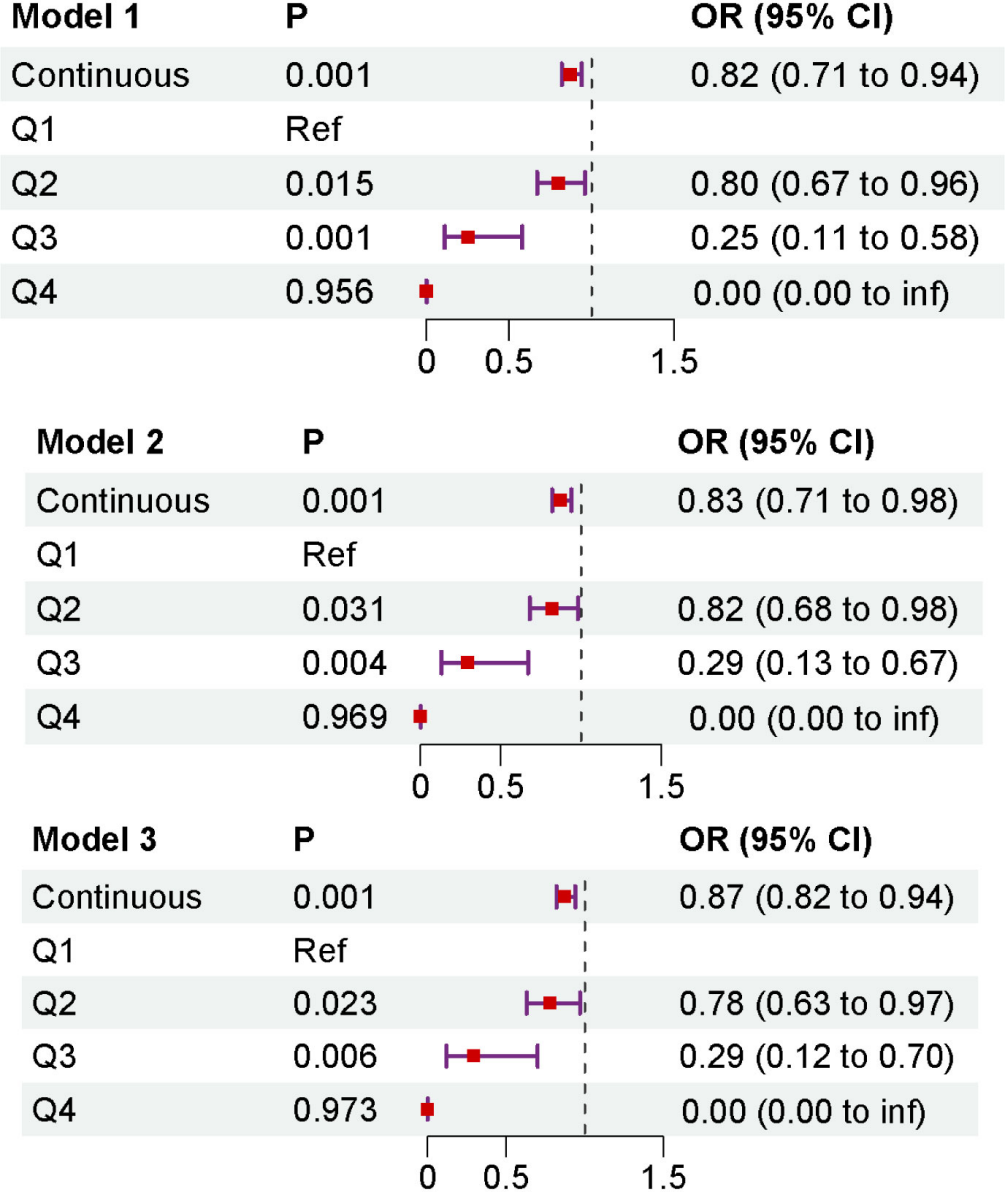
The association between DI-GM and sarcopenia was analyzed by logistic regression. Model 1: no covariate adjustment; Model 2: adjusted for age, sex and race; Model 3: adjusted for age, sex, education level, race, PIR, marital status, BMI, smoking status, alcohol drinking, diabetes, hypertension, and CVD.

The logistic regression analysis revealed that a higher DI-GM index was associated with a gradual reduction in the risk of sarcopenia. In the unadjusted model, DI-GM demonstrated a significant inverse association with sarcopenia risk (OR = 0.82, 95% CI: 0.71–0.94). After adjusting for all covariates, this inverse association remained statistically significant (OR = 0.87, 95% CI: 0.82–0.94, p < 0.001), (**Figure 2**).

For sensitivity analysis, DI-GM was categorized into multiple groups. Notably, across all models, the Q3 group (8 ≤ DI-GM < 11) exhibited the lowest odds ratio among the four quartiles. Compared to Q1, the risk of sarcopenia in Q3 was significantly reduced by 61%. However, this association was not significant in the highest quartile (Q4).

### Nonlinear trends in DI-GM and sarcopenia

**Figure 3:**
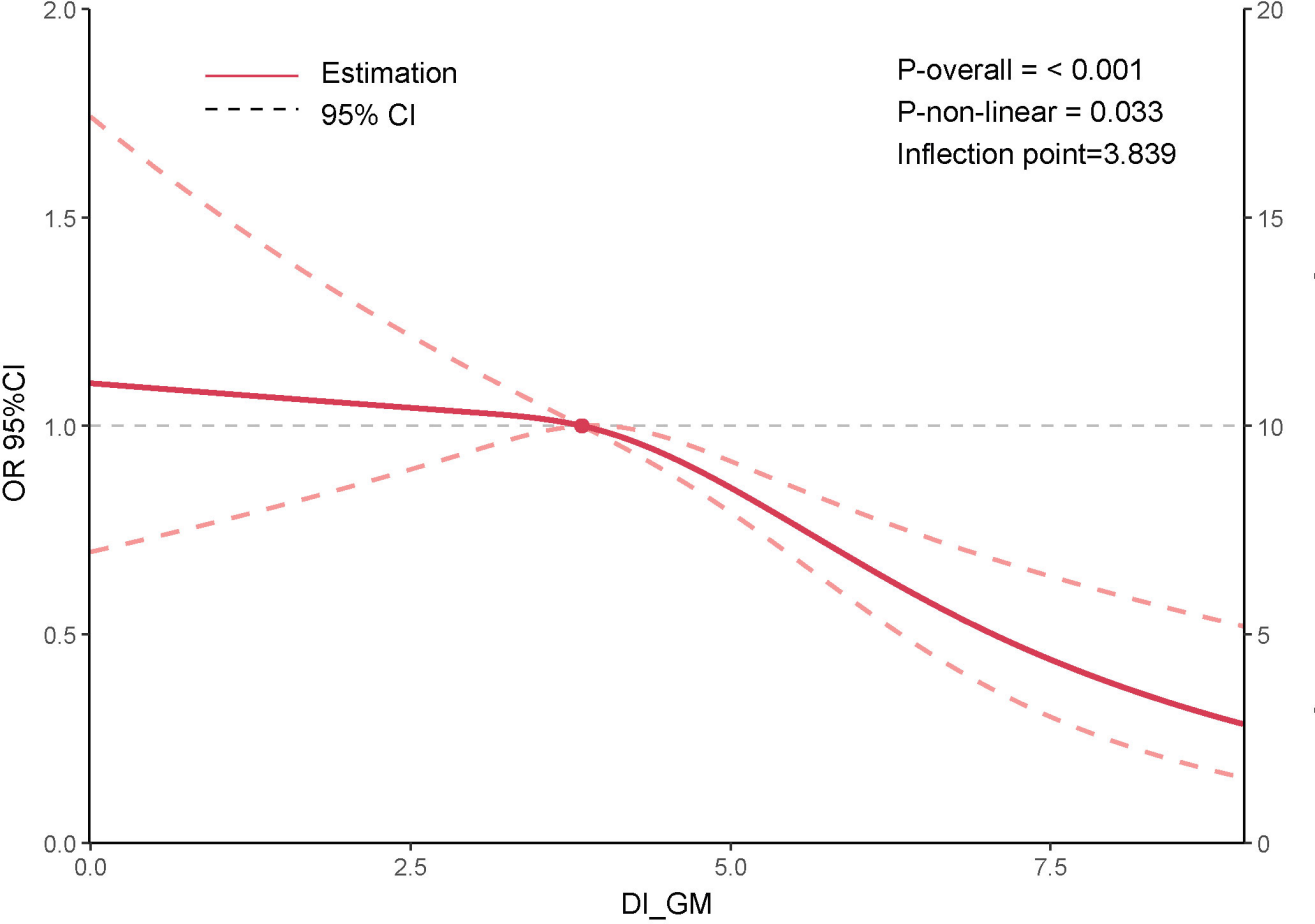
Association between DI-GM and sarcopenia.

This study utilized a RCS curve to examine the potential nonlinear association between DI-GM and sarcopenia. After adjusting for age, sex, race/ethnicity, education level, marital status, PIR, BMI, smoking status, alcohol consumption, diabetes, hypertension, CVD, and physical activity, the results indicated that a higher DI-GM score was associated with a gradual reduction in sarcopenia risk, (**Figure 3**).

Furthermore, a significant nonlinear relationship between DI-GM and sarcopenia was observed (P-non-linear = 0.033). Notably, when DI-GM exceeded the threshold of 3.839, the decline in sarcopenia risk became more pronounced, **Supplementary Table S2**.

### Stratified analysis

**Figure 4:**
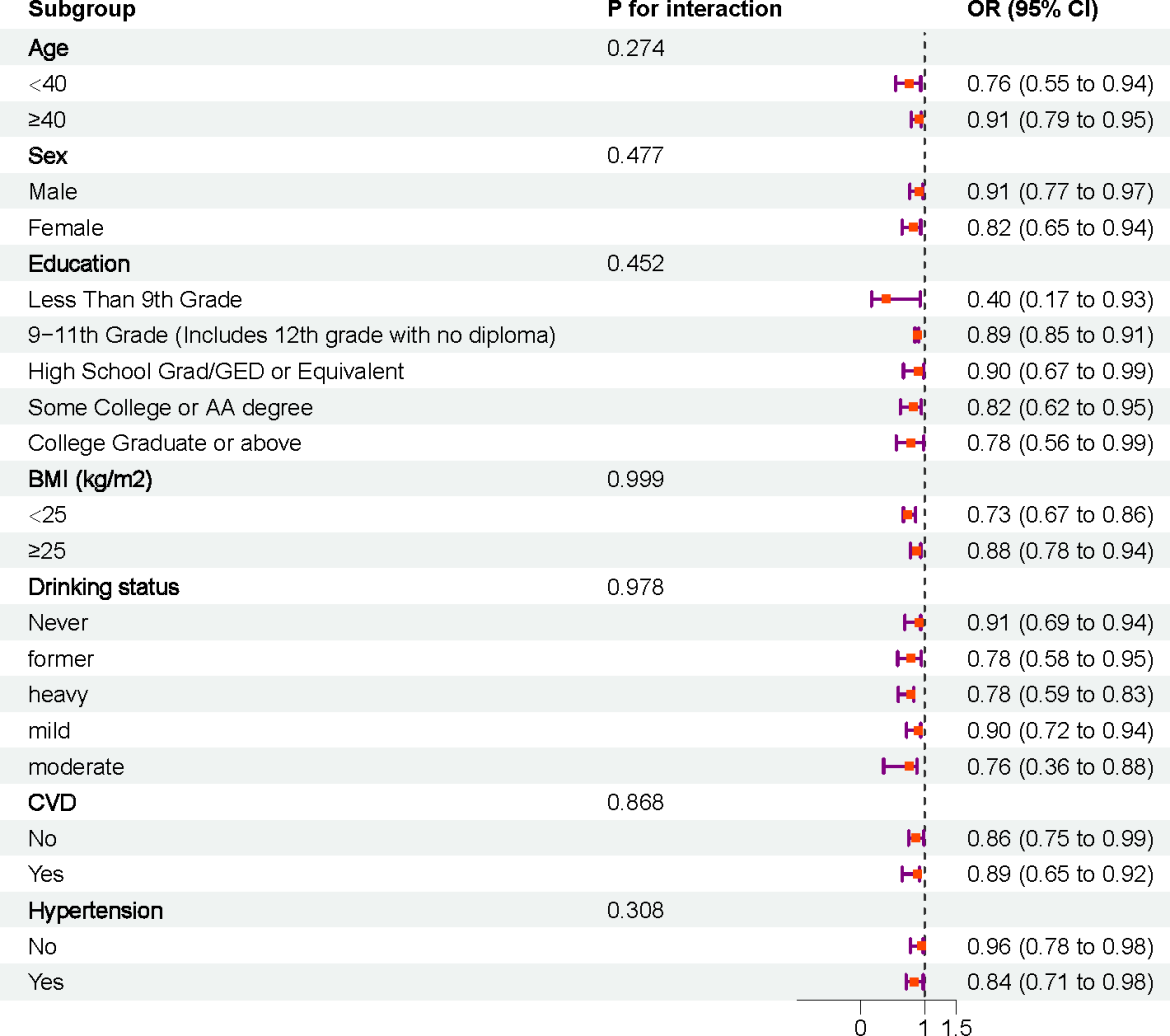
Subgroup analysis.

Stratified analyses and interaction tests were performed based on age, sex, education, BMI, alcohol consumption, smoking, CVD, and hypertension, (**Figure 4**). The results showed that the association between DI-GM and sarcopenia was consistent across different subgroups, and no significant interactions were observed.

### Establishment of the predictive nomogram

**Figure 5:**
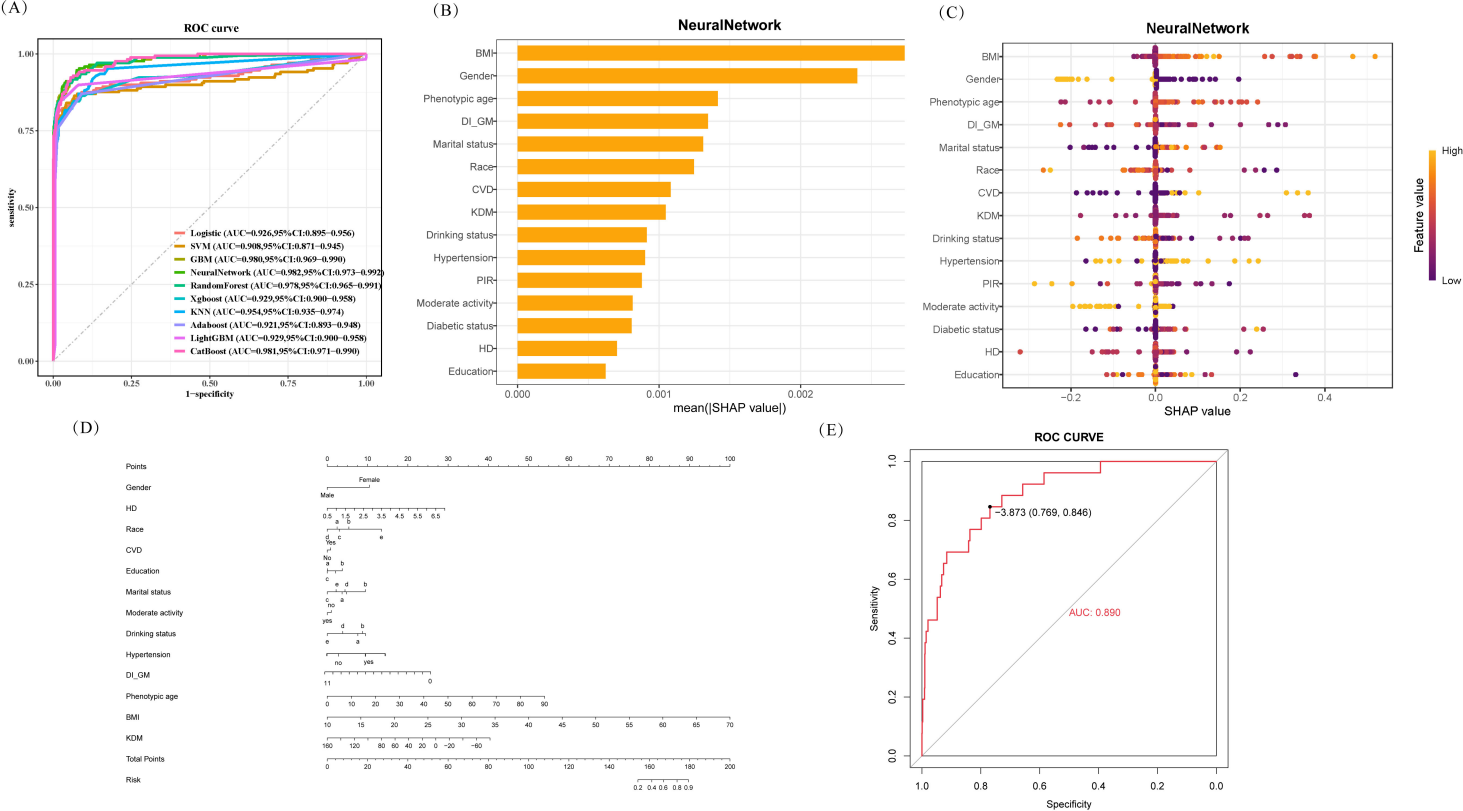
Construction of nomogram model. **(A)** ROC curves for multiple models, highlighting the best‐performing classifier via AUC. **(B)** Bar graph of mean SHAP values in the Neural Network model, reflecting each feature’s relative importance. **(C)** SHAP summary plot of feature contributions; each dot denotes an observation, with color representing high (yellow) or low (purple) feature values. **(D)** Nomogram integrating key SHAP‐identified predictors, where each variable’s point contribution sums to yield a total risk score. **(E)** ROC curve in the validation set (AUC = 0.890), demonstrating the nomogram’s strong discrimination.

To further evaluate the discriminative ability of DI-GM and other predictive factors in sarcopenia, we developed a nomogram, (**Figure 5**). First, we compared the performance of multiple predictive models. Using the ROC curve and confusion matrix from the validation set, we assessed model performance based on the Youden index, AUC, accuracy, and F1 score. Ultimately, the Neural Network model was identified as the optimal classifier, **Supplementary Table S3**.

Next, we conducted Shapley Additive Explanations analysis, including beeswarm plots and feature importance rankings, to identify the most influential predictive variables. These key variables were then incorporated into the nomogram. Specifically, each predictor was mapped to a corresponding score on the nomogram, and the cumulative score was projected onto the total score scale, providing an intuitive visual estimation of sarcopenia risk.

Finally, we evaluated the model’s discriminative ability using the ROC curve, which yielded an AUC of 0.8895 (95% CI: 0.8299–0.9492). This result indicates that the constructed nomogram exhibits strong predictive performance for sarcopenia.

### Mediation analysis

**Figure 6:**
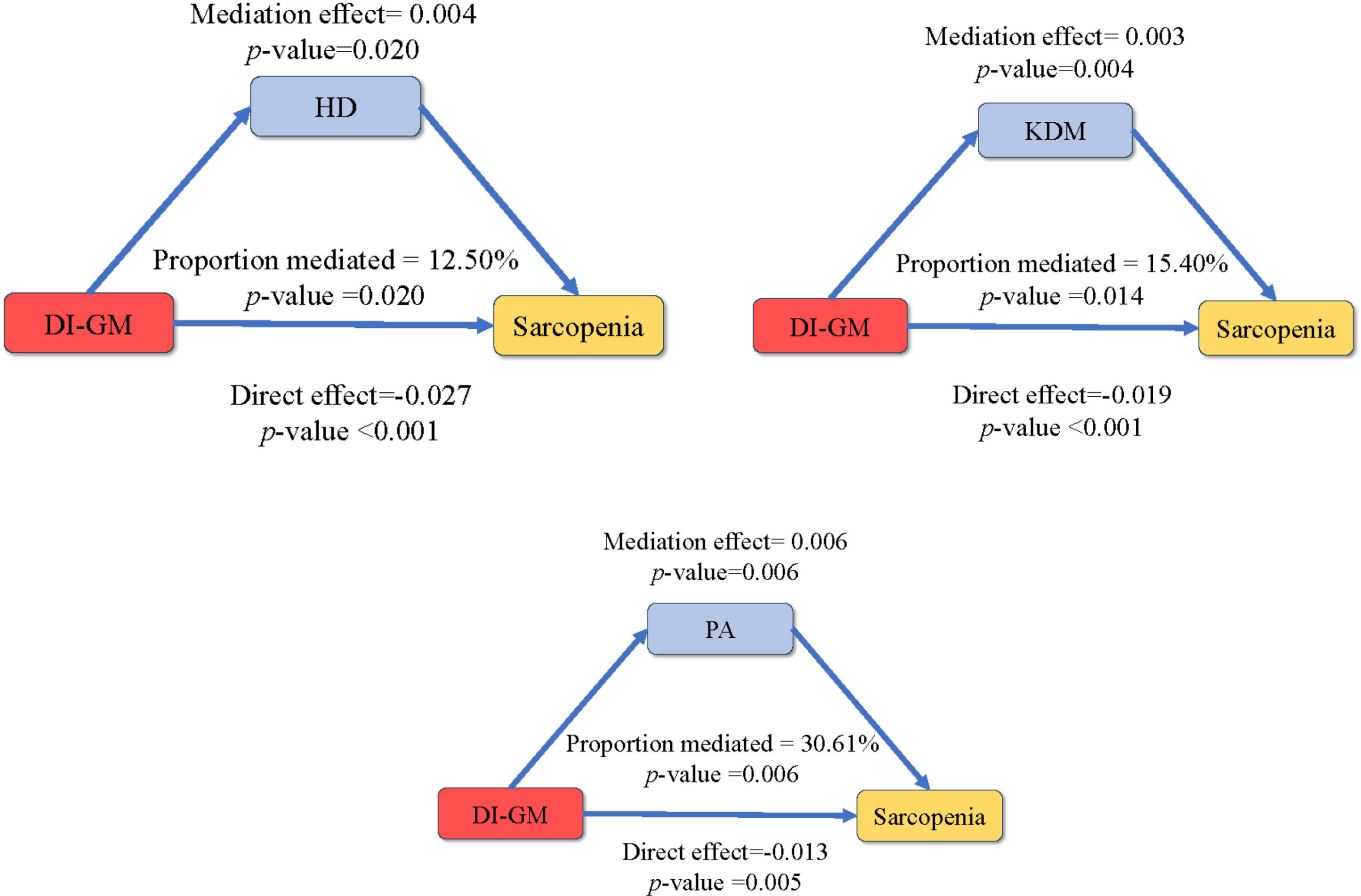
Biological age-mediated association between DI-GM and sarcopenia. The model was adjusted for sex, education, race, PIR, marital, BMI, smoking, drinking, diabetes, hypertension, CVD.

This study utilized mediation analysis to explore the potential mediating roles of three biological age-related indicators (PhenoAge, HD, and KDM) in the association between DI-GM and sarcopenia, (**Figure 6**). The results showed that the mediating effects of HD, KDM, and PhenoAge on the association between DI-GM and sarcopenia were 0.004 (p = 0.020), 0.003 (p = 0.004), and -0.006 (p = 0.006), respectively, with mediation proportions of 12.5%, 15.4%, and 30.6%, **Supplementary Table S4**. Moreover, higher phenotypic age, HD, and KDM were significantly associated with an increased risk of sarcopenia. The odds ratios were 1.06 (95% CI: 1.04–1.09, p < 0.001) for PA, 1.41 (95% CI: 1.05–1.90, p = 0.021) for HD, and 1.02 (95% CI: 1.01–1.03, p < 0.001) for KDM, **Supplementary Table S5**.

## Discussion

This study is the first to demonstrate the association between a gut microbiota-friendly diet and sarcopenia. The results showed that higher DI-GM scores were associated with a lower risk of sarcopenia. Logistic regression models indicated that participants in higher DI-GM score groups had significantly lower risks of sarcopenia. The RCS curve further revealed a nonlinear association between DI-GM and sarcopenia. Mediation analysis indicated that the three biological age indicators played varying degrees of mediating roles in the association between DI-GM and sarcopenia, with PhenoAge exhibiting the highest mediation proportion.

The Diet-Microbiota-Gut-Muscle Axis serves as a critical framework linking diet to muscle health, emphasizing how dietary factors modulate gut microbiota composition and function, thereby influencing muscle metabolism and overall health (25). The DI-GM, a dietary metric reflecting the influence of gut microbiota, encompasses SCFAs, bioactive peptides, and lactate, all of which contribute to microbial homeostasis. These components collectively act on the gut micro-ecosystem and ultimately regulate the microbiota-gut-muscle axis. Several studies have demonstrated that modulating gut microbiota through bacterial depletion, fecal transplantation, and dietary supplementation can directly impact muscle phenotypes (26). Probiotics, prebiotics, SCFAs, and bacterial metabolites are emerging as potential therapeutic strategies to enhance muscle mass and physical performance. Building on these findings, this study explores the potential role of a high DI-GM dietary pattern in mitigating sarcopenia risk.

In recent years, the impact of diet on gut microbiota and muscle health has garnered increasing research interest. Multiple studies have investigated the relationship between gut microbial diversity and muscle health, identifying Bifidobacterium adolescentis, Faecalibacterium, and Lactobacillus as positively correlated with muscle mass, whereas Bacteroidetes, Clostridiales, and Enterobacteriales exhibit a negative correlation (27–31). Despite the potential of microbial supplements in ameliorating sarcopenia, only three randomized controlled trials have assessed their efficacy, and results remain inconclusive (7, 8, 32). The first study (N = 60) administered the prebiotic Darmocare Pre^®^ (fructooligosaccharides + oligofructose) to an elderly population, reporting significant improvements in grip strength and fatigue levels (p < 0.05), although no significant effects were observed in functional, cognitive, or sleep quality metrics (32). The second study (N = 54) investigated Lactobacillus plantarum TWK10 supplementation in a young, healthy population, demonstrating muscle mass gains and dose-dependent anti-fatigue effects, suggesting that probiotics may enhance muscle function via energy metabolism regulation and exercise endurance improvement (7). However, the third study (N = 17) tested a synbiotic intervention (fructooligosaccharides + Lactobacillus + Bifidobacterium) in elderly participants and found no significant changes in lean body mass or muscle strength, though minor improvements in hydration status were noted in individual analyses (8). This finding indicate that, in certain cases, synbiotics may not effectively improve sarcopenia-related parameters.

In contrast, this study employs DI-GM, a comprehensive dietary index, to investigate the relationship between gut microbiota and sarcopenia from a dietary pattern perspective, rather than focusing solely on microbial supplementation. This approach enables an assessment of long-term dietary patterns and their influence on gut microbiota diversity and its potential protective role in muscle health. DI-GM incorporates 14 beneficial and detrimental dietary components, including fermented dairy products, whole grains, soy, broccoli, cranberries, and green tea, which support gut microbiota balance, while processed meats, red meat, and high-fat diets are associated with microbiota dysbiosis. Furthermore, DI-GM evaluates multiple gut microbiota markers, such as SCFAs and microbial diversity, providing a comprehensive assessment of the gut-muscle axis and its role in diet-microbiota-muscle interactions.

In this study, we found that a higher DI-GM score was significantly associated with a reduced risk of sarcopenia. This association remained robust after adjusting for all confounding variables (OR = 0.87, 95% CI: 0.82–0.94, p < 0.001). Notably, among all models, the Q3 group (8 ≤ DI-GM < 11) exhibited the strongest protective effect, with a 61% reduction in sarcopenia risk compared to Q1. However, this association was not significant in the highest quartile (Q4). This pattern is consistent with previous findings on the Gut-Muscle Axis. From a biological perspective, a higher DI-GM score indicates a diet rich in fermented foods, polyphenols, and dietary fiber, which serve as prebiotic substrates. These substrates promote short-chain fatty acid (SCFA) production and enhance gut barrier function (33). SCFAs, particularly butyrate and propionate, not only serve as energy substrates but also inhibit the NF-κB pathway and downregulate pro-inflammatory cytokines (IL-6, TNF-α, and CRP) (34). This mechanism mitigates the adverse effects of chronic inflammation on muscle fiber synthesis and repair. Additionally, gut microbiota-derived metabolites activate key signaling pathways, including mTOR and AMPK, in the liver and muscle tissues. The mTOR pathway plays a crucial role in muscle protein synthesis regulation, whereas the AMPK pathway is essential for energy sensing and mitochondrial biogenesis (35).

However, an excessively high DI-GM score (Q4) did not confer additional protective effects, which may be due to multiple factors. First, a high-fiber or predominantly plant-based diet may lead to insufficient intake of essential nutrients such as protein, iron, and zinc, or result in micronutrient imbalances. Moreover, anti-nutritional factors (e.g., phytates and oxalates) may further reduce the bioavailability of minerals and protein synthesis substrates. Additionally, aging-related factors, such as increased insulin resistance and mitochondrial dysfunction, may reduce the responsiveness of aging muscle to exogenous or gut microbiota-derived metabolites. Consequently, even a theoretically optimal dietary pattern may not further lower sarcopenia risk in older adults. This threshold effect was also evident in our RCS analysis, which demonstrated that the protective effect of DI-GM became more pronounced once the score exceeded approximately 3.839. However, no additional linear benefit was observed with further increases in DI-GM (P-non-linear = 0.033).This “threshold effect” may result from the dysregulated interaction of multiple molecular pathways. First, aging and insulin resistance lead to abnormal activation or suppression of key regulators, including FoxO3, MyoD, and muscle atrophy-specific ubiquitin ligases (e.g., Atrogin-1/MAFbx and MuRF1) (36, 37). This disruption disturbs the balance between muscle protein synthesis and degradation, accelerating muscle loss (38). Second, as mitochondrial function declines, muscle cells exhibit reduced efficiency in utilizing energy substrates, such as short-chain fatty acids (SCFAs) (39). This impairment limits their ability to maintain optimal oxidative phosphorylation rates, thereby weakening the protective effects of gut microbiota-derived metabolites on muscle metabolism (40). Third, in older individuals, gut microbiota homeostasis is inherently more fragile (41). Even with sufficient intake of fermentable fibers, polyphenols, and other prebiotic substrates, it remains challenging to sustain an optimal microbial balance over time (42, 43). Additionally, chronic inflammation (inflammaging) may further exacerbate neuromuscular junction (NMJ) dysfunction, blocking or weakening gut microbiota-mediated signaling pathways involved in muscle protein synthesis (44).In summary, this study underscores the importance of balanced dietary optimization. When DI-GM scores are low, indicating an inadequate dietary structure, increasing prebiotic intake and diversifying nutrient sources can enhance the production of beneficial metabolites, such as SCFAs. This process supports muscle synthesis and repair. Conversely, when DI-GM scores are excessively high, inadequate protein intake, micronutrient imbalances, or adherence to extreme dietary patterns may lead to unintended adverse effects.

Another notable finding of this study is that biological aging indicators (PA, KDM, and HD) mediate the relationship between DI-GM and sarcopenia, suggesting that multiple physiological aging pathways contribute to muscle mass maintenance. These three indicators evaluate the interaction between diet, gut microbiota, and muscle health from different perspectives, including systemic aging, metabolic homeostasis, and inflammatory dysregulation. This finding suggests that DI-GM may influence sarcopenia risk by modulating biological age. PA reflects overall physiological aging, encompassing inflammation, metabolic function, liver and kidney health, and cardiovascular status. Zhang et al. reported that a diet rich in fiber, polyphenols, and fermented foods reduces oxidative stress and improves vascular function (45). These effects are partly mediated by the modulation of inflammatory cytokines, such as IL-6 and TNF-α, which influence biological age (46). The mediating role of PA in the association between higher DI-GM scores and reduced sarcopenia risk suggests that dietary patterns may slow systemic aging and delay the onset of sarcopenia. This finding aligns with the accelerated aging hypothesis, which proposes that dietary components (e.g., ω-3 fatty acids, polyphenols, and micronutrients) significantly affect DNA methylation patterns (DNAm) associated with aging.

KDM, a key indicator of metabolic homeostasis and tissue repair capacity, has been shown to predict all-cause mortality and chronic disease risk (47). The observed mediating role of KDM in the association between DI-GM and sarcopenia suggests that dietary patterns influence muscle health by optimizing metabolic stability and enhancing tissue repair mechanisms. Previous studies indicate that dietary antioxidants (e.g., SCFAs and polyphenols) regulate KDM by reducing DNA methylation damage and increasing mitochondrial activity (48). Furthermore, unique dietary components within DI-GM, such as fermented dairy products, soybean oligosaccharides, sucrose, and chlorogenic acid, may influence KDM by improving insulin sensitivity, reducing oxidative stress, and promoting muscle protein synthesis, thereby lowering sarcopenia risk. HD predominantly influences physiological health through its role in inflammatory response (CRP, IL-6, TNF-α), oxidative stress, and metabolic disorders (49). This study confirmed the mediating role of HD in the relationship between DI-GM and sarcopenia. Specifically, a high DI-GM dietary pattern may reduce HD by modulating gut microbiota, decreasing intestinal permeability, and mitigating LPS-induced chronic inflammation, thereby preserving muscle function. Multiple studies have demonstrated that harmful bacterial metabolites, such as indoxyl sulfate and lipopolysaccharides (LPS), activate the PI3K/AKT, NF-κB, and MAPK (p38, JNK, ERK) signaling pathways, upregulating the expression of E3 ubiquitin ligases (Atrogin-1 and MuRF1) (50). This process accelerates muscle degradation and chronic inflammation. Additionally, gut microbiota dysbiosis leads to the downregulation of muscle growth-related genes (e.g., IGF-1, MyoD, and Myogenin) and an increase in myostatin (a negative regulator of muscle mass), further limiting muscle maintenance during aging (51). These findings align with our results, suggesting that higher DI-GM scores may help maintain muscle metabolic homeostasis by alleviating chronic inflammation and supporting mitochondrial and neuromuscular junction function.

## Limitations

First, as a cross-sectional study, it does not allow for determining a causal relationship between DI-GM and sarcopenia. Second, as with most studies, the influence of unmeasured variables or unknown confounders cannot be entirely ruled out. Furthermore, since the NHANES database only collected whole-body DXA data from 2007 to 2020, and the 2019–2020 pandemic significantly altered dietary habits, physical activity levels, and metabolic health, our analysis was primarily restricted to the 2007–2018 period. Finally, dietary recall data may be subject to recall bias.

## Conclusion

DI-GM, an index reflecting the relationship between diet and gut microbiota diversity, was found to have a nonlinear association with sarcopenia. An increase in DI-GM scores was significantly associated with a reduced risk of sarcopenia. Notably, the three biological age-related indicators (KDM, PhenoAge, and HD) partially mediated the association between DI-GM and sarcopenia, with PhenoAge exhibiting the highest mediation proportion at 30.6%. Given the limited and inconsistent results of human studies on treating sarcopenia with microbial supplements, focusing on dietary habits may be a more optimal approach for research. Future studies should incorporate gut microbiome data to evaluate the practicality and applicability of the findings.

## Data Availability

The original contributions presented in the study are included in the article/**Supplementary Material**. Further inquiries can be directed to the corresponding author.
