# Supplementary material for: The mediating role of biological age in the association between dietary index for gut microbiota and sarcopenia
Source: Front Immunol. 2025 Mar 21;16:1552525. doi: 10.3389/fimmu.2025.1552525 (PMC11968661; doi:10.3389/fimmu.2025.1552525)
Supplement: Supplementary file 1 [file Table1.docx]

Supplementary Table 1. Study population baseline table

|  | Total | Q1 [0, 4] | Q2 [5, 8] | Q3 [8, 10] | Q4 [11, 14] | P-value |
| --- | --- | --- | --- | --- | --- | --- |
| Variable |  | 1523 | 4304 | 208 | 3 |  |
| Age (years) | 36.58 ± 13.78 | 36.25 ± 13.84 | 36.67 ± 13.66 | 39.82 ± 12.03 | 41.00 ± 19.67 | 0.005 |
| PIR | 2.45 ± 1.68 | 2.20 ± 1.60 | 2.51 ± 1.69 | 3.08 ± 1.68 | 5.00 ± 0.00 | <0.001 |
| BMI (kg/m2) | 28.38 ± 7.35 | 29.16 ± 7.76 | 28.26 ± 7.19 | 26.99 ± 6.95 | 27.03 ± 6.62 | <0.001 |
| Waist(cm) | 95.75 ± 18.31 | 97.54 ± 18.66 | 95.54 ± 18.04 | 92.58 ± 17.62 | 94.27 ± 19.31 | <0.001 |
| Sex, n (%) |  |  |  |  |  | 0.004 |
| Male | 3433 (54.72%) | 869 (57.06%) | 2329 (54.11%) | 92 (44.23%) | 2 (66.67%) |  |
| Female | 2841 (45.28%) | 654 (42.94%) | 1975 (45.89%) | 116 (55.77%) | 1 (33.33%) |  |
| Race, n (%) |  |  |  |  |  | <0.001 |
| Mexican American | 549(9.10%) | 145(9.56%) | 385(8.95%) | 12(5.72%) | 0 (0.00%) |  |
| Other Hispanic | 362(6.21%) | 109(7.17%) | 234(5.45%) | 9(4.24%) | 0 (0.00%) |  |
| Non-Hispanic White | 3966(65.69%) | 952(62.54%) | 2935(68.20%) | 151(72.38%) | 2(84.59%) |  |
| Non-Hispanic Black | 670(11.11%) | 208(13.68%) | 394(9.15%) | 9(4.41%) | 0 (0.00%) |  |
| Other Race - Including Multi-Racial | 476(7.89%) | 107(7.05%) | 355(8.25%) | 28(13.25%) | 1(15.41%) |  |
| Education, n (%) |  |  |  |  |  | <0.001 |
| Less Than 9th Grade | 248 (4.32%) | 56 (4.03%) | 171 (4.31%) | 4 (1.98%) | 0 (0.00%) |  |
| 9-11th Grade (Includes 12th grade with no diploma) | 723 (12.60%) | 210 (15.13%) | 475 (11.98%) | 14 (6.93%) | 0 (0.00%) |  |
| High School Grad/GED or Equivalent | 1230 (21.44%) | 375 (27.02%) | 789 (19.90%) | 31 (15.35%) | 0 (0.00%) |  |
| Some College or AA degree | 1916 (33.40%) | 474 (34.15%) | 1320 (33.30%) | 62 (30.69%) | 1 (33.33%) |  |
| College Graduate or above | 1620 (28.24%) | 273 (19.67%) | 1209 (30.50%) | 91 (45.05%) | 2 (66.67%) |  |
| Marital status, n (%) |  |  |  |  |  | <0.001 |
| Married | 2745 (47.85%) | 582 (41.93%) | 1946 (49.09%) | 116 (57.43%) | 1 (33.33%) |  |
| Widowed | 73 (1.27%) | 31 (2.23%) | 39 (0.98%) | 3 (1.49%) | 0 (0.00%) |  |
| Divorced | 572 (9.97%) | 137 (9.87%) | 401 (10.12%) | 15 (7.43%) | 0 (0.00%) |  |
| Separated | 202 (3.52%) | 58 (4.18%) | 126 (3.18%) | 9 (4.46%) | 0 (0.00%) |  |
| Never married | 1588 (27.68%) | 409 (29.47%) | 1087 (27.42%) | 47 (23.27%) | 2 (66.67%) |  |
| Living with partner | 557 (9.71%) | 171 (12.32%) | 365 (9.21%) | 12 (5.94%) | 0 (0.00%) |  |
| Moderate activity, n (%) |  |  |  |  |  | <0.001 |
| Yes | 2633 (45.37%) | 567 (40.33%) | 1875 (46.75%) | 121 (59.90%) | 1 (33.33%) |  |
| No | 3171 (54.63%) | 839 (59.67%) | 2136 (53.25%) | 81 (40.10%) | 2 (66.67%) |  |
| Drinking status, n (%) |  |  |  |  |  | <0.001 |
| Never | 714 (12.45%) | 164 (11.79%) | 489 (12.34%) | 26 (12.87%) | 0 (0.00%) |  |
| former | 668 (11.64%) | 177 (12.72%) | 456 (11.51%) | 13 (6.44%) | 0 (0.00%) |  |
| heavy | 1472 (25.66%) | 403 (28.97%) | 998 (25.19%) | 35 (17.33%) | 0 (0.00%) |  |
| mild | 1831 (31.92%) | 419 (30.12%) | 1266 (31.95%) | 86 (42.57%) | 2 (66.67%) |  |
| moderate | 1052 (18.34%) | 228 (16.39%) | 753 (19.01%) | 42 (20.79%) | 1 (33.33%) |  |
| Smoking status, n (%) |  |  |  |  |  | 0.001 |
| never | 3374 (58.80%) | 785 (56.52%) | 2367 (59.71%) | 119 (58.91%) | 2 (66.67%) |  |
| former | 968 (16.87%) | 220 (15.84%) | 667 (16.83%) | 49 (24.26%) | 0 (0.00%) |  |
| now | 1396 (24.33%) | 384 (27.65%) | 930 (23.46%) | 34 (16.83%) | 1 (33.33%) |  |
| Diabetic status, n (%) |  |  |  |  |  | 0.156 |
| no | 5269 (83.98%) | 1264 (82.99%) | 3638 (84.53%) | 170 (81.73%) | 3 (100.00%) |  |
| DM | 627 (9.99%) | 167 (10.97%) | 412 (9.57%) | 26 (12.50%) | 0 (0.00%) |  |
| IFG | 166 (2.65%) | 52 (3.41%) | 104 (2.42%) | 3 (1.44%) | 0 (0.00%) |  |
| IGT | 212 (3.38%) | 40 (2.63%) | 150 (3.49%) | 9 (4.33%) | 0 (0.00%) |  |
| CVD, n (%) |  |  |  |  |  | 0.38 |
| No | 5506 (95.97%) | 1321 (95.17%) | 3814 (96.22%) | 193 (95.54%) | 3 (100.00%) |  |
| Yes | 231 (4.03%) | 67 (4.83%) | 150 (3.78%) | 9 (4.46%) | 0 (0.00%) |  |
| Hypertension, n (%) |  |  |  |  |  | 0.12 |
| No | 4596 (73.36%) | 1083 (71.20%) | 3174 (73.81%) | 158 (75.96%) | 3 (100.00%) |  |
| Yes | 1669 (26.64%) | 438 (28.80%) | 1126 (26.19%) | 50 (24.04%) | 0 (0.00%) |  |
| Sarcopenia, n (%) |  |  |  |  |  | 0.005 |
| No | 5651 (90.07%) | 1363 (89.49%) | 3905 (90.73%) | 202 (97.12%) | 3 (100.00%) |  |
| Yes | 623 (9.93%) | 160 (10.51%) | 399 (9.27%) | 6 (2.88%) | 0 (0.00%) |  |
| HD | 3.33 (3.29 ,3.37) | 3.32 (3.27 ,3.37) | 3.33 (3.29 ,3.37) | 3.45 (3.31 ,3.58) | 3.65 (3.35 ,3.95) | 0.0607 |
| KDM | 5.07 (4.32 ,5.83) | 5.00 (4.17 ,5.83) | 5.16 (4.20 ,6.12) | 5.11 (1.71 ,8.50) | -9.28 (-14.86 ,-3.70) | 0.0005 |
| PHENOAGE | 37.46 (36.74 ,38.17) | 36.85 (36.23 ,37.47) | 37.97 (37.05 ,38.89) | 38.47 (35.88 ,41.06) | 38.36 (32.67 ,44.06) | 0.0499 |
| Albumin, g/L | 43.13 ± 3.28 | 42.97 ± 3.32 | 43.28 ± 3.23 | 43.37 ± 3.14 | 42.33 ± 3.06 | 0.006 |
| Creatinine, umol/L | 76.82 ± 32.51 | 77.71 ± 38.07 | 76.23 ± 26.44 | 72.31 ± 15.17 | 74.55 ± 11.37 | 0.047 |
| Alkaline phosphatase, U/L | 65.09 ± 20.85 | 66.14 ± 21.07 | 64.19 ± 20.58 | 62.35 ± 20.67 | 51.67 ± 10.41 | 0.001 |
| Total cholesterol, mg/dL | 191.19 ± 41.56 | 191.18 ± 40.84 | 191.21 ± 42.44 | 190.87 ± 40.64 | 208.00 ± 6.24 | 0.018 |
| Uric acid, umol/L | 319.41 ± 82.03 | 321.65 ± 83.36 | 318.67 ± 81.05 | 298.97 ± 73.53 | 285.50 ± 47.19 | 0.002 |
| WBC count (1000 cells/uL) | 7.22 ± 2.18 | 7.30 ± 2.21 | 7.14 ± 2.14 | 7.17 ± 2.17 | 6.50 ± 1.39 | 0.048 |
| Lymphocyte percent, % | 31.30 ± 8.21 | 31.24 ± 8.26 | 31.37 ± 8.21 | 31.27 ± 7.44 | 39.03 ± 11.00 | 0.0395 |
| Mean cell volume, fL | 88.86 ± 5.80 | 88.81 ± 5.87 | 88.88 ± 5.77 | 89.15 ± 5.29 | 89.80 ± 3.94 | 0.045 |
| Systolic blood pressure | 118.90 ± 14.89 | 119.40 ± 15.05 | 118.57 ± 14.81 | 116.16 ± 13.35 | 113.78 ± 16.78 | 0.01 |
| Blood urea nitrogen, mg/dL | 11.80 ± 4.39 | 11.79 ± 4.60 | 11.83 ± 4.20 | 11.70 ± 3.91 | 13.33 ± 2.89 | 0.008 |
| Glycohemoglobin, % | 5.60 ± 1.04 | 5.64 ± 1.13 | 5.55 ± 0.91 | 5.63 ± 1.09 | 5.17 ± 0.23 | 0.012 |

Supplementary Table 2. Threshold effect analysis

|  | OR (95%CI) | P |
| --- | --- | --- |
| Continuous | 0.87 (0.82, 0.94) | 0.001 |
| Inflection point（K） | 3.839 |  |
| <K | 0.93 (0.84, 0.98) | 0.158 |
| >K | 0.77 (0.65, 0.92) | 0.004 |
| Log likelihood ratio |  | 0.033 |

The model was adjusted for age, sex, education, race, PIR, marital, BMI, smoking, drinking, diabetes, hypertension, CVD

Supplementary Table 3. Comparison of Classification Metrics among Different Machine Learning Models

| Model | Threshold | Accuracy | Sensitivity | Specificity | Precision | F1 |
| --- | --- | --- | --- | --- | --- | --- |
| Logistic | 0.357 | 0.970 | 0.817 | 0.985 | 0.852 | 0.834 |
| SVM | 0.248 | 0.947 | 0.840 | 0.958 | 0.673 | 0.747 |
| GBM | 0.061 | 0.930 | 0.929 | 0.931 | 0.579 | 0.714 |
| NeuralNetwork | 0.057 | 0.927 | 0.947 | 0.925 | 0.565 | 0.708 |
| RandomForest | 0.168 | 0.954 | 0.899 | 0.960 | 0.697 | 0.786 |
| Xgboost | 0.496 | 0.926 | 0.846 | 0.934 | 0.570 | 0.681 |
| KNN | 0.043 | 0.879 | 0.917 | 0.875 | 0.431 | 0.586 |
| Adaboost | 0.128 | 0.906 | 0.870 | 0.909 | 0.497 | 0.632 |
| LightGBM | 0.073 | 0.964 | 0.846 | 0.976 | 0.786 | 0.815 |
| CatBoost | 0.523 | 0.943 | 0.923 | 0.945 | 0.632 | 0.750 |

Supplementary Table 4. Mediation Analysis

| HD | Estimate | 95% CI lower | 95% CI upper | P-value |
| --- | --- | --- | --- | --- |
| Total effect | -0.023 | -0.038 | -0.007 | <0.0001 |
| Mediation effect (average) | 0.004 | 0.001 | 0.007 | 0.020 |
| Direct effect (average) | -0.027 | -0.039 | -0.014 | <0.0001 |
| Propotion mediated (average) | 0.125 | 0.021 | 0.263 | 0.020 |
| KDM |  |  |  |  |
| Total effect | -0.016 | -0.029 | -0.004 | 0.010 |
| Mediation effect (average) | 0.003 | 0.001 | 0.005 | 0.004 |
| Direct effect (average) | -0.019 | -0.031 | -0.006 | <0.0001 |
| Propotion mediated (average) | 0.154 | 0.035 | 0.701 | 0.014 |
| PA |  |  |  |  |
| Total effect | -0.007 | -0.024 | 0.010 | <0.0001 |
| Mediation effect (average) | 0.006 | 0.002 | 0.010 | 0.006 |
| Direct effect (average) | -0.013 | -0.026 | 0.000 | 0.005 |
| Propotion mediated (average) | 0.306 | 0.104 | 0.986 | 0.006 |

Note. The model was adjusted for sex, education, race, PIR, marital, BMI, smoking, drinking, diabetes, hypertension, CVD

Supplementary Table 5. Multivariable logistic regression results for associations between three biological aging measures (HD, KDM, and Phenotypic Age) and sarcopenia

| Exposure | Model 1 | Model 2 | Model 3 |
| --- | --- | --- | --- |
| HD | OR (95% CI)/P value | OR (95% CI)/P value | OR (95% CI)/P value |
| Continuous | 1.47 (1.14, 1.89) 0.0030 | 1.77 (1.33, 2.34) <0.0001 | 1.41 (1.05, 1.90) 0.0212 |
| Q1 | Reference | Reference | Reference |
| Q2 | 2.75 (1.21, 6.22) 0.0154 | 3.07 (1.35, 6.99) 0.0076 | 2.92 (1.27, 6.71) 0.0115 |
| Q3 | 3.25 (1.46, 7.27) 0.0040 | 4.54 (1.99, 10.40) 0.0003 | 3.62 (1.57, 8.34) 0.0025 |
| Q4 | 4.28 (1.95, 9.43) 0.0003 | 7.15 (3.05, 16.76) <0.0001 | 4.58 (1.91, 10.98) 0.0007 |
| KDM |  |  |  |
| Continuous | 1.02 (1.02, 1.03) <0.0001 | 1.03 (1.02, 1.04) <0.0001 | 1.02 (1.01, 1.03) <0.0001 |
| Q1 | Reference | Reference | Reference |
| Q2 | 1.35 (0.54, 3.40) 0.5230 | 1.57 (0.62, 3.96) 0.3423 | 1.45 (0.57, 3.71) 0.4370 |
| Q3 | 1.81 (0.75, 4.34) 0.1855 | 2.29 (0.94, 5.55) 0.0676 | 1.90 (0.77, 4.71) 0.1637 |
| Q4 | 4.30 (1.93, 9.60) 0.0004 | 5.70 (2.51, 12.96) <0.0001 | 3.31 (1.39, 7.91) 0.0070 |
| Phenotypic age |  |  |  |
| Continuous | 1.08 (1.06, 1.10) <0.0001 | 1.08 (1.06, 1.10) <0.0001 | 1.06 (1.04, 1.09) <0.0001 |
| Q1 | Reference | Reference | Reference |
| Q2 | 1.13 (1.03, 1.31) <0.0001 | 1.11 (1.02, 1.28) <0.0001 | 1.09 (1.04, 1.17) <0.0001 |
| Q3 | 1.10 (1.01, 1.27) <0.0001 | 1.19 (1.01, 1.25) <0.0001 | 1.07 (1.03, 1.15) <0.0001 |
| Q4 | 1.74 (1.47, 2.14) 0.3791 | 1.68 (1.43, 2.06) 0.2228 | 1.22 (1.08, 1.44) <0.0001 |

Note. Model 1: no covariate adjustment; Model 2: adjusted for sex and race; Model 3: adjusted for sex, education level, race, PIR, marital status, BMI, smoking status, alcohol drinking, diabetes, hypertension, and CVD.
